# miR-21-3p Regulates Influenza A Virus Replication by Targeting Histone Deacetylase-8

**DOI:** 10.3389/fcimb.2018.00175

**Published:** 2018-05-25

**Authors:** Binghui Xia, Jiansheng Lu, Rong Wang, Zhixin Yang, Xiaowei Zhou, Peitang Huang

**Affiliations:** Laboratory of Protein Engineering, Beijing Institute of Biotechnology, Beijing, China

**Keywords:** influenza A virus, H1N1, H5N1, miR-21-3p, HDAC8, virus replication

## Abstract

Influenza A virus (IAV) is responsible for severe morbidity and mortality in animals and humans worldwide. miRNAs are a class of small noncoding single-stranded RNA molecules that can negatively regulate gene expression and play important roles in virus-host interaction. However, the roles of miRNAs in IAV infection are still not fully understood. Here, we profiled the cellular miRNAs of A549 cells infected with A/goose/Jilin/hb/2003 (H5N1) and a comparison A/Beijing/501/2009 (H1N1). miRNA microarray and quantitative PCR analysis showed that several miRNAs were differentially expressed in A549 cells during IAV infection. Subsequently, we demonstrated that IAV replication was essential for the regulation of these miRNAs, and bioinformatic analysis revealed that the targets of these miRNAs affected biological processes relevant to IAV replication. Specifically, miR-21-3p was found to be down-regulated in IAV-infected A549 cells and selected for further detailed analysis. Target prediction and functional study illustrated that miR-21-3p repressed the expression of HDAC8 by targeting its 3′UTR. Furthermore, we confirmed miR-21-3p could promote virus replication, which was similar to the result of knocking down HDAC8, indicating that miR-21-3p promoted IAV replication by suppressing HDAC8 expression. Altogether, our results suggest a potential host defense against IAV through down-regulation of miR-21-3p.

## Introduction

Influenza A virus (IAV), a member of the family *Orthomyxoviridae*, is responsible for severe morbidity and mortality in animals and humans worldwide (Wright et al., 2013). At the beginning of the twentieth century, the 1918 flu was estimated to have caused approximately 50 million human deaths (Taubenberger and Morens, 2006). In 2009, a previously undescribed IAV H1N1 emerged in North America and spread to other countries worldwide with a high transmissibility but relative low fatality rate, causing the first pandemic in the twenty-first century (Smith et al., 2009). More seriously, in 1997, the highly pathogenic H5N1 subtype was first observed to be spread from poultry to humans and cause deaths in Hong Kong (Subbarao et al., 1998). Up to December 2017, more than 860 confirmed cases of human infections with the H5N1 virus, including 454 deaths, have been reported to the World Health Organization with a high fatality rate of approximately 53% (http://www.who.int/influenza/). Subsequently, a novel highly pathogenic avian IAV H7N9 emerged in eastern China in 2013 (Gao et al., 2013). To date, the case fatality rate of H7N9 virus infection has reached as high as 39% with at least 612 deaths from 1565 confirmed cases (http://www.who.int/influenza/). Vaccination is currently considered the best strategy for protection from IAV infection (Pica and Palese, 2013). However, the antigenic drift and antigenic shift of IAV allow for the possibility of reassortment and escape from current various treatments, creating a public health concern (Layne et al., 2009; Herfst et al., 2012). Thus, to prevent IAV infection, the development of new effective prophylactic or therapeutic strategies are urgently needed.

miRNAs are a class of 18–23 nt noncoding single-stranded RNA molecules that can negatively regulate gene expression through binding to the 3′ untranslated regions (UTRs) of their target genes. miRNAs play important roles in a wide range of physiological and pathological processes, such as development, proliferation, differentiation, apoptosis, cancer and viral infections (Bushati and Cohen, 2007; Skalsky and Cullen, 2010). Recently, several studies indicated that miRNAs could be involved in the process of IAV infection. For example, a few miRNAs, such as let-7c, miR-2911, miR-323, miR-491, and miR-654 had been found to inhibit IAV replication by directly targeting viral genes (Song et al., 2010; Ma et al., 2012; Zhou et al., 2015). In addition, Nakamura et al. (2015) found that IAV-induced expression of GalNAc transferase 3 (GALNT3) enhanced viral replication through down-regulating miR-17-3p and miR-221.

During miRNA biogenesis,miRNA genes are transcribed into primary miRNAs and then cleaved into miRNA duplexes composed of a guide strand and a passenger strand (also known as miRNA and miRNA^*^) by Drosha and Dicer. Generally, the guide strand is selectively incorporated by an Argonaute (AGO) protein into the miRNA-induced silencing complex (miRISC), while the passenger strand is considered to be degraded due to its lower stability (Guo and Lu, 2010). Recent studies showed that many miRNA^*^ species are relatively rich and play important roles in various processes (Jin et al., 2011; Byrd et al., 2012; Yan et al., 2015; Báez-Vega et al., 2016). However, to date, few studies have reported on the function of miRNA^*^ species during IAV infection.

In this study, miRNA microarray analysis was performed in A549 cells infected with A/goose/Jilin/hb/2003 (H5N1) and a comparison A/Beijing/501/2009 (H1N1). We identified several miRNAs, including miR-21-3p, which were differentially expressed in IAV-infected A549 cells, and IAV replication was found to be essential for the regulation of these miRNAs. Subsequently, Gene Ontology (GO) and pathway analysis revealed that the targets of these miRNAs could affect biological processes relevant to IAV replication. A down-regulated miRNA, miR-21-3p, was selected for further detailed analysis. Based on target prediction and functional analysis, we demonstrated that miR-21-3p could repress the expression of HDAC8 by targeting its 3′UTR and promote IAV replication *in vitro*. These findings suggest that the host may resist IAV through down-regulation of miR-21-3p.

## Materials and methods

### Cell culture and virus

A549 and MDCK cells were obtained from the American Type Culture Collection (ATCC, USA), and 293FT cells were obtained from Invitrogen (USA). These cells were propagated in Dulbecco′s Modified Eagle′s Medium (DMEM; Life Technologies, USA) supplemented with 10% fetal bovine serum (FBS; HyClone, USA) at 37°C in a 5% CO_2_ incubator.

The IAV strain A/Beijing/501/2009 (H1N1) was kindly provided by Beijing Institute of Microbiology and Epidemiology, and the IAV strain A/goose/Jilin/hb/2003 (H5N1) was kindly provided by Prof. Hualan Chen (Harbin Veterinary Research Institute, Chinese Academy of Agricultural Sciences). The two IAV strains were propagated and titrated to determine the 50% tissue culture infection dose (TCID50) in MDCK cells. The TCID50 per ml values were determined using the Reed and Muench statistical method (Reed and Muench, 1938). UV-H1N1 and UV-H5N1 were generated by UV irradiation of H1N1 and H5N1 for 15 min. All experiments with IAVs were performed in a Biosafety Level 3 laboratory (Beijing Institute of Microbiology and Epidemiology).

### Viral infection

For viral infection, A549 cells were washed with phosphate-buffered saline (PBS) and subsequently infected with IAVs at the multiplicity of infection (MOI) of 0.1 or 5 in a minimal volume of DMEM supplemented 0.5 mg/ml TPCK-treated trypsin (Sigma–Aldrich, USA) and 0.3% bovine serum albumin (Sangon, China) (infection medium). After 1 h, cells were incubated with fresh infection medium at 37°C for the indicated times.

### MicroRNA microarray analysis

A549 cells were cultured in 24-well plates in triplicate and identically processed by removing their growth medium, followed by three washes with PBS before adding infection medium containing H1N1 or H5N1 virus at a MOI of 5 for 8 and 24 h. As a control, simulation of the procedure for virus infection was performed on another triplicate group of uninfected cells. At 8 and 24 h post-infection (hpi), total RNAs of the uninfected and infected A549 cells were extracted by using the mirVana miRNA Isolation Kit (Ambion, USA) and checked for an RIN number by using an Agilent Bioanalyzer 2100 (Agilent Technologies, USA). MicroRNA microarray analysis were performed by Shanghai Biochip. The differences between samples were analyzed by using unsupervised analysis (SAS system, Shanghai Biochip, China). Only those miRNAs with the absolute fold change ≥1.5 or ≤ 0.67 and *P*-value < 0.05 were considered significant. The miRNA data have been uploaded in NCBI′s Gene Expression Omnibus (GEO) database and are accessible through GEO Series accession number GSE96857.

### Bioinformatic analyses

miRNA sequences were retrieved from the miRBase sequence database. The potential miRNA target sites were predicted by using two algorithm-based software programs, TargetScan version 6.2 (http://www.targetscan.org) and MicroCosm version 5.0 (http://www.ebi.ac.uk/Enright-srv/microcosm/cgibin/targets/v5/search.pl). Pathway analysis and GO term identification were performed with DAVID (Huang da et al., 2009).

### Real-time quantitative PCR (qPCR)

Total RNA, containing miRNA, was extracted with the mirVana miRNA Isolation Kit (Ambion) following the manufacturer′s instructions. Total RNA was reverse transcribed using the First-Strand cDNA Synthesis Kit (Takara, Japan) with an oligo-dT primer for mRNA or a specific stem-loop primer (Bulge-Loop^TM^ miRNA qPCR Primers from RiboBio, China). Real-time qPCR was performed on the IQ5 Real-Time PCR Detection System (Bio-Rad, USA), using SYBR Green qPCR SuperMix (Transgen Biotech, China). Relative expression levels were calculated by applying the 2^−ΔΔ*Ct*^ threshold method (Livak and Schmittgen, 2001) using *GAPDH* for mRNA and *U6* snRNA for miRNA.

For analysis of influenza virus *NP* vRNA in treated cells, total RNA, containing viral RNA, was reverse transcribed with a universal primer (Uni-12, 5′-AGCGAAAGCAGG-3′) complementary to the conserved end of the influenza genome. Influenza virus genes were subsequently assayed on the IQ5 Real-Time PCR Detection System (Bio-Rad) with specific *NP* gene primers. Data were normalized by the level of *GAPDH* expression in each sample as described above. The sequences of primers used for real-time qPCR are contained in Table S1 or available upon request.

### Plasmids and reagents

The 3′UTRs of predicted target genes were PCR-amplified from A549 cDNA and cloned into the luciferase reporter vector pMIR-report (Ambion). *HDAC8* 3′UTR target site mutations were constructed using a fast mutagenesis system (Transgen, China). The sequences of the *HDAC8* 3′UTR construct used in this experiment were changed from 5′-TGGTGTT-3′ to 5′-ACCACAA-3′. The ORF of *HDAC8* genes was PCR-amplified from A549 cDNA and cloned into the mammalian expression vector pCMV-Tag2B (Stratagene). All constructs were confirmed by sequencing. All sequences of primers used for plasmids construction are contained in Table S1 or available upon request.

Chemically synthesized miRNA mimics and miRNA inhibitors (miR-141-3p, miR-200c-3p, miR-21-3p, and miR-29b-1-5p) were purchased from RiboBio (China). miRNA inhibitors were single-strand RNA molecules with a 2′-O-methyl modification, the sequence of which were complementary to the miRNAs. Negative controls (RiboBio) were transfected as internal controls. siRNA duplexes were synthesized and purified by RiboBio. Briefly, three siRNAs targeting the human *HDAC8* gene were designed, and the most effective siRNA (siHDAC8-1) identified by real-time qPCR was applied for further experiments. Cells were seeded in 24-well plates and incubated for 24 h at 37°C, and then the siRNA (25 nmol), miRNA mimics (50 nmol), miRNA inhibitors (100 nmol) and negative control were transfected into the cells by using Lipofectamine 2000 (Life Technologies, USA). The antibody against HDAC8 was purchased from Santa Cruz Biotechnology (USA). The antibody against β-actin and poly(I:C) were purchased from Sigma-Aldrich.

### Luciferase reporter assay

293FT cells (1 × 10^4^) were plated in 96-well plates and transfected with a mixture of the luciferase reporter plasmid and the pRL-TK plasmid together with miRNA mimics. Cells were collected at 24–36 h after transfection, and luciferase activity was measured with a Dual-Luciferase assay (Promega, USA) following the manufacturer′s protocol. Reporter gene activity was determined by normalizing firefly luciferase activity to Renilla luciferase activity.

### Western blot

Whole cells were harvested in total protein extraction buffer (TPEB; Transgen Biotech, China) containing a protease inhibitor cocktail (Roche, Germany), and the protein concentration was measured by the BCA protein assay (Cwbiotech, China). Equal amounts of protein were separated by SDS-PAGE and transferred to PVDF membranes (Millipore, USA). The membranes were incubated with various primary antibodies, followed by a horseradish peroxidase (HRP)-conjugated secondary antibody. The signals were detected using an Immobilon Western Chemiluminescent HRP Substrate kit (Thermo Fisher, USA). The quantification of Western blot analysis was performed by using Quantity One software, and the protein expression levels were normalized to β-actin levels.

### Statistical analysis

All experiments were repeated at least three times. Data were expressed as means ± standard deviation (SD). Statistical significance was determined by two-tailed Student′s *t-*test for two comparisons or analysis of variance (ANOVA) with the Newman-Keuls test for multiple comparisons using GraphPad Prism 5.0 (GraphPad Software). A *P*-value below 0.05 was considered statistically significant.

## Results

### Differential miRNA expression during IAV infection of human cells

**Figure 1:**
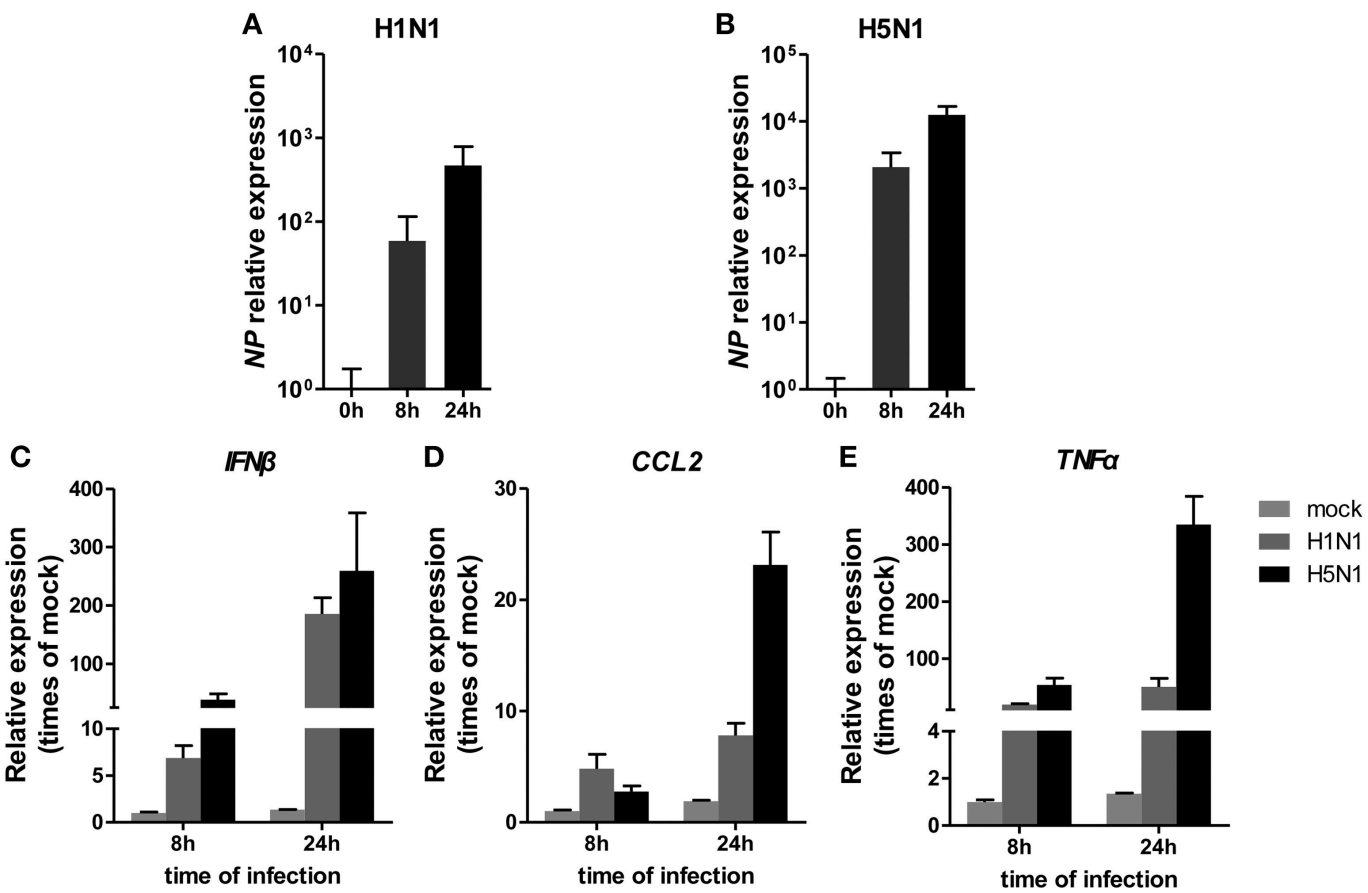
Cellular response to IAV infection. A549 cells were uninfected or infected with A/Beijing/501/2009 (H1N1) and A/goose/Jilin/hb/2003 (H5N1) at a MOI of 5.0 for the indicated times. The relative abundance of the IAV *NP* gene **(A,B)**, *IFN*β **(C)**, *CCL*2 **(D)**, and *TNF*α **(E)** was measured by real-time qPCR and normalized to that of *GAPDH*. Data are the mean ± SD of three independent experiments.

**Figure 2:**
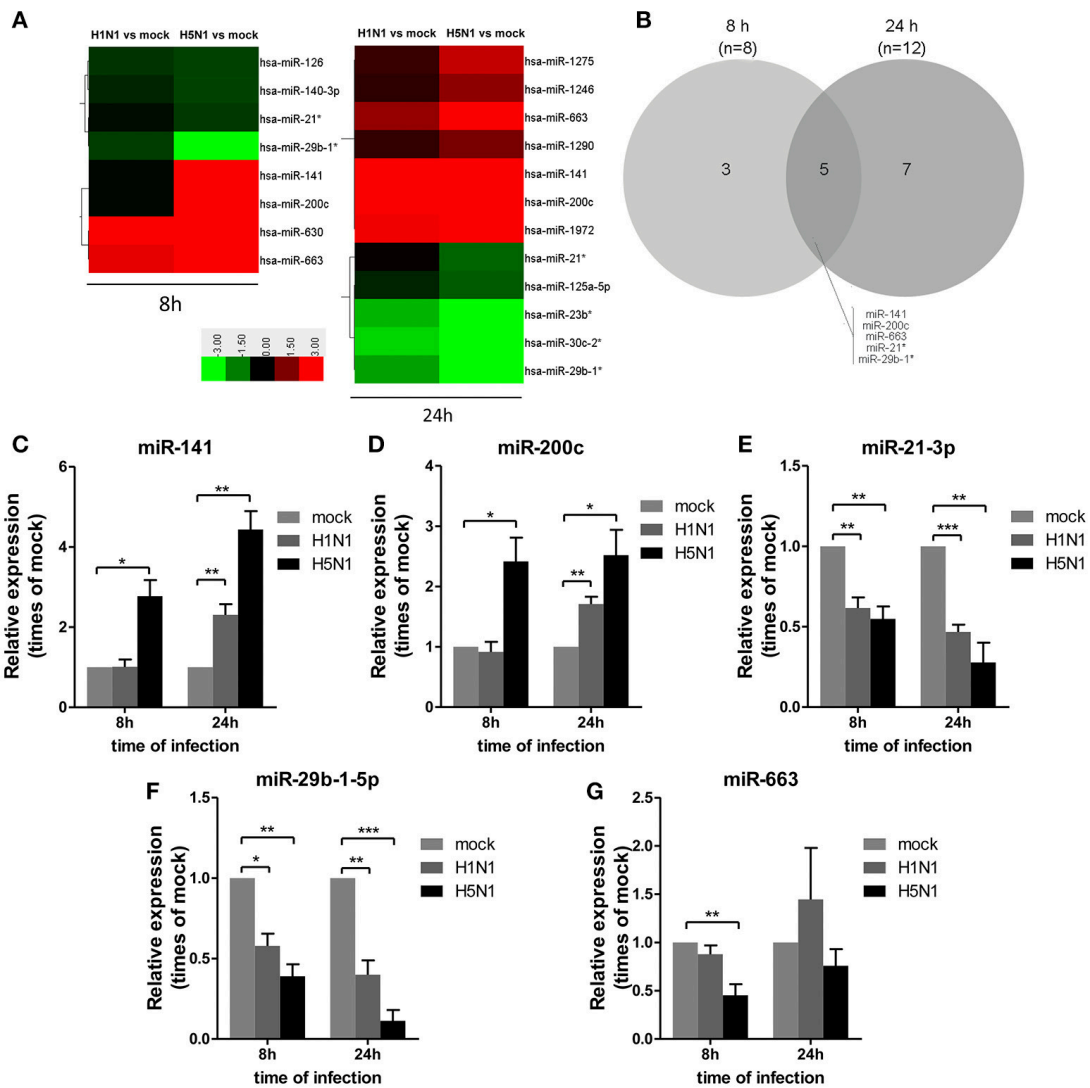
Differential miRNA expression during IAV infection. A549 cells were uninfected or infected with A/Beijing/501/2009 (H1N1) and A/goose/Jilin/hb/2003 (H5N1) at a MOI of 5.0 for the indicated times. **(A)** Heat map representing miRNAs that were differentially expressed during IAV infection at 8 hpi (left) and 24 hpi (right). Colors indicate the log2 ratios of IAV-infected group vs. uninfected control following the specified scale. **(B)** Venn diagram of differentially expressed miRNAs during IAV infection at 8 hpi vs. 24 hpi. **(C–G)** miRNAs from **(A)** were confirmed at the indicated time points by real-time qPCR analysis. Expression levels of indicated miRNAs were normalized to that of *U6* and relative to mock-infected cells. Data are the mean ± SD from four independent experiments. ^*^P < 0.05, ^**^P < 0.01 and ^***^P < 0.001 by one-way ANOVA.

To investigate miRNA expression profiles during IAV infection, two IAV strains, A/Beijing/501/2009 (H1N1) and A/goose/Jilin/hb/2003 (H5N1), were used to infect the human alveolar epithelial cell line A549. Total RNAs were isolated at 8 and 24 hpi. In parallel, RNA samples from mock-infected cells were prepared as control. First, to verify the host cell response to IAV infection, the levels of antiviral mRNAs, such as *IFN*β*, CCL*2, and *TNF*α, and the transcript for the IAV nucleoprotein were examined. All detected genes were significantly induced by IAV infection (Figure 1). Additionally, the cytopathic effect (CPE) of infected cells was also observed to ensure efficient infection (Figure S1). Together, these results indicated permissive and productive infection of A549 cells by IAV. Subsequently, we performed the miRNA expression profile of A549 cells infected with H1N1 and H5N1 as described in the materials and methods section. The differentially expressed miRNAs were identified using the cut-off criteria of absolute fold change ≥ 1.5 or ≤ 0.67 and *t*-test *P* < 0.05 following H1N1 infection or H5N1 infection relative to mock-treated cells (Figure 2A). Following this method, a total of 8 and 10 dysregulated miRNAs were identified at 8 and 24 hpi, respectively (Figure 2B). A subset of five miRNAs was both dysregulated at 8 h and 24 h post-infection, including miR-141, miR-200c, miR-21^*^ (also known as miR-21-3p), miR-29b-1^*^ (also known as miR-29b-1-5p) and miR-663. However, the fold changes occurred in H1N1 infection were much lower than that in H5N1 infection.

To verify differential miRNA expression identified by microarray profiling in IAV-infected cells, the microarray data were further confirmed by real-time qPCR (Figures 2C–G). miR-141 and miR-200c were both increased at 8 and 24 hpi in H5N1-infected cells, while they were only increased at 24 hpi in H1N1-infected cells (Figures 2C,D). Moreover, miR-21-3p and miR-29b-1-5p were decreased at 8 hpi and 24 hpi in H1N1- or H5N1-infected cells (Figures 2E,F). These real-time qPCR results were generally consistent with the microarray results. Unfortunately, miR-663 was not found to show any significant changes from 8 to 24 hpi (Figure 2G). Together, these results supported the conclusion that miR-141 and miR-200c were up-regulated, while miR-21-3p and miR-29b-1-5p were down-regulated during IAV infection of human cells.

### IAV replication is essential for regulating expression of miRNAs including miR-21-3p

**Figure 3:**
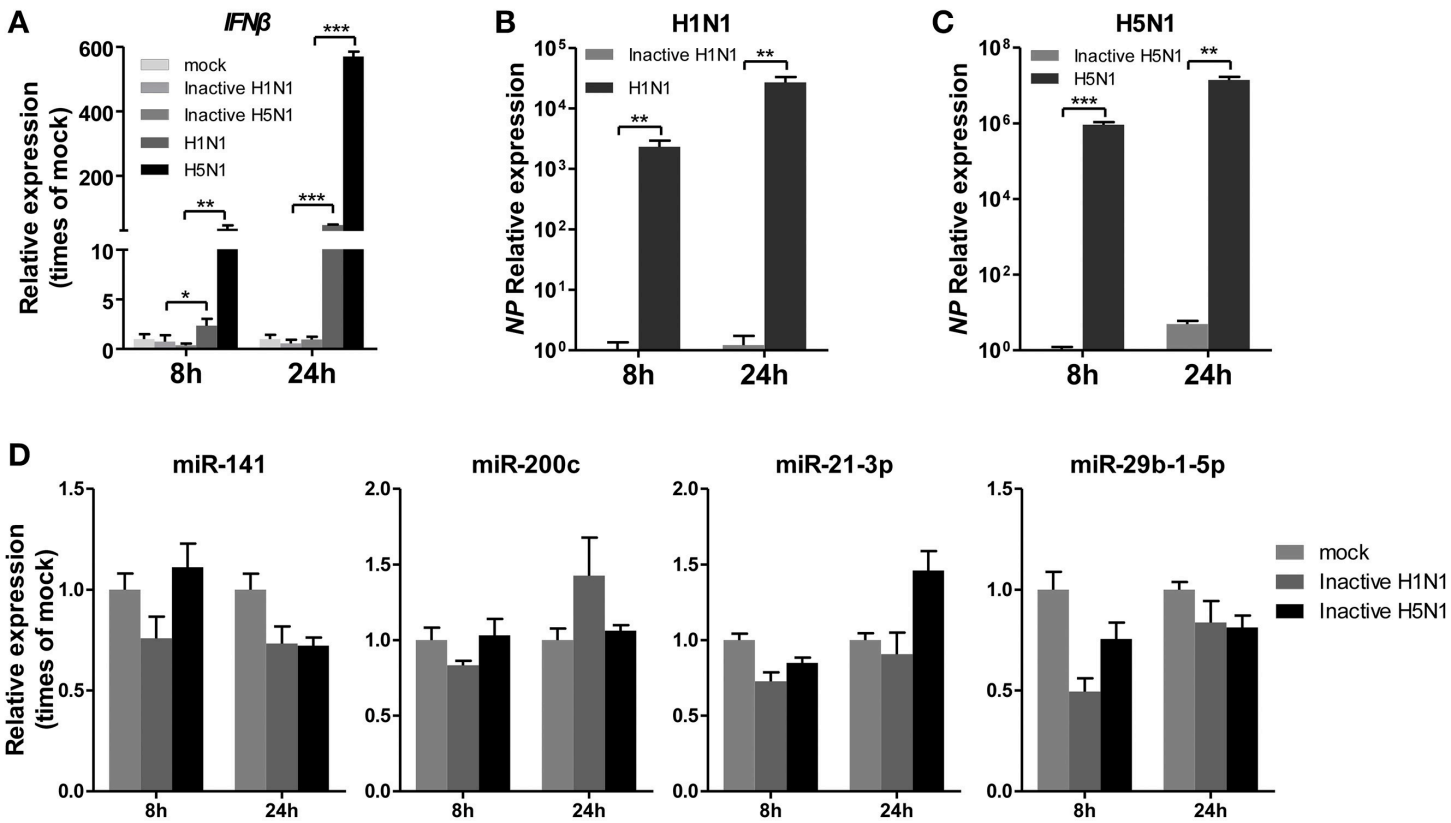
UV-irradiated IAV does not regulate expression of miRNAs. A549 cells were inoculated with UV-irradiated H1N1 and H5N1 at a MOI of 5.0 for the indicated times. **(A–C)** Expression levels of *IFN*β and the IAV *NP* gene were detected by real-time qPCR analysis to confirm the replication of the inoculated virus. Samples were normalized to expression of *GAPDH*. **(D)** Expression levels of miR-141, miR-200c, miR-21-3p, and miR-29b-1-5p were determined via real-time qPCR. Samples were normalized to expression of *U6* snRNA and relative to mock-infected cells. Data are the mean ± SD of triplicate samples from a single experiment and are representative of two independent experiments. ^*^P < 0.05, ^**^P < 0.01 and ^***^P < 0.001 by one-way ANOVA or *t*-test.

To determine whether IAV replication in cells is essential for the regulation of miRNA expression, A549 cells were infected with mock- or UV-irradiated H1N1 and H5N1 virus in parallel with H1N1 and H5N1, and the expression of miRNAs was observed at 8 and 24 hpi. As a control, the expression of *IFN*β was detected and found to be slightly up-regulated in inactive H1N1 and H5N1-inoculated cells (Figure 3A). Real-time qPCR analysis of the *NP* gene also revealed that the inactive H1N1 and H5N1 could not replicate in cells during the 8–24 hpi period (Figures 3B,C). Under this condition, miR-141 and miR-200c were not up-regulated, and miR-21-3p and miR-29b-1-5p were only slightly down-regulated at 8 hpi (Figure 3D). These results showed that inactive IAV was not essential for the regulation of miRNAs, including miR-141, miR-200c, miR-21-3p, and miR-29b-1-5p.

**Figure 4:**
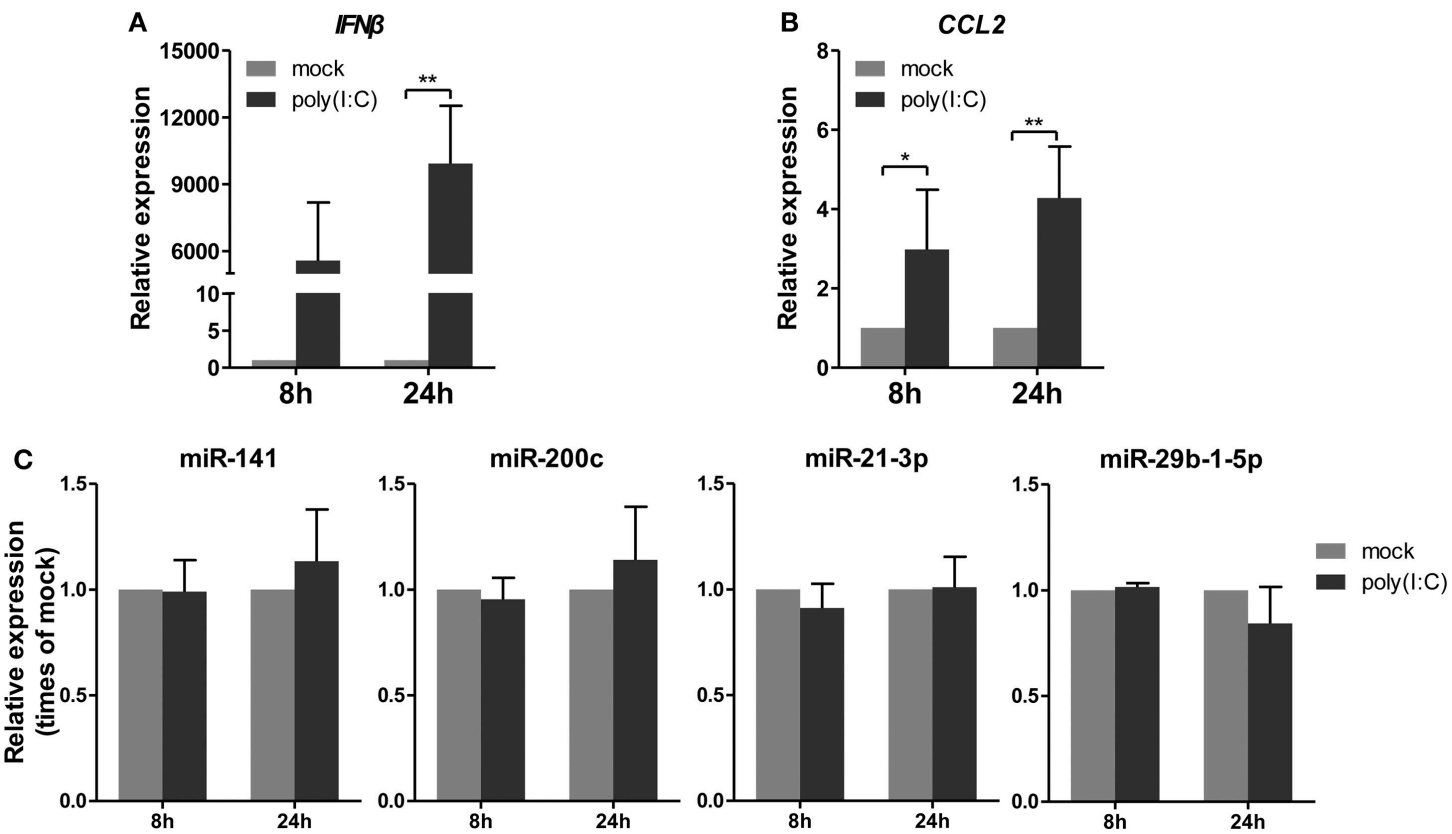
Poly(I:C) does not regulate expression of miRNAs. A549 cells were stimulated with 10 μg/ml poly(I:C) or mock treated for the indicated times. **(A,B)** Expression levels of *IFN*β and *CCL2* were detected by real-time qPCR analysis to verify response to poly(I:C) stimulation. Samples were normalized to expression of *GAPDH* and relative to mock-infected cells. **(C)** Expression of miR-141, miR-200c, miR-21-3p, and miR-29b-1-5p was determined via real-time qPCR. Samples were normalized to expression of *U6* snRNA and relative to mock-infected cells. Data are the mean ± SD from four independent experiments. ^*^P < 0.05 and ^**^P < 0.01 vs. mock infection by *t*-test.

Subsequently, a synthetic mimetic of viral double-stranded RNA, poly(I:C), which can activate the antiviral pattern recognition receptors TLR3 and RIG-I/MDA5 and mimic IAV infection (Alexopoulou et al., 2001; Traynor et al., 2004), was transfected into A549 cells. The expression levels of *IFN*β and *CCL2* were measured to confirm the efficacy of the poly(I:C) transfection (Figures 4A,B). Under this condition, the four miRNAs remained constant or slightly changed. These results indicated that poly(I:C) and its induction signaling were not essential for the regulation of miRNAs (Figure 4C). Together, these results indicated that only live IAV, not inactive IAV or poly(I:C), could regulate the four miRNAs expression, including miR-21-3p.

### IAV-regulated miRNAs affect biological processes relevant to IAV replication

**Figure 5:**
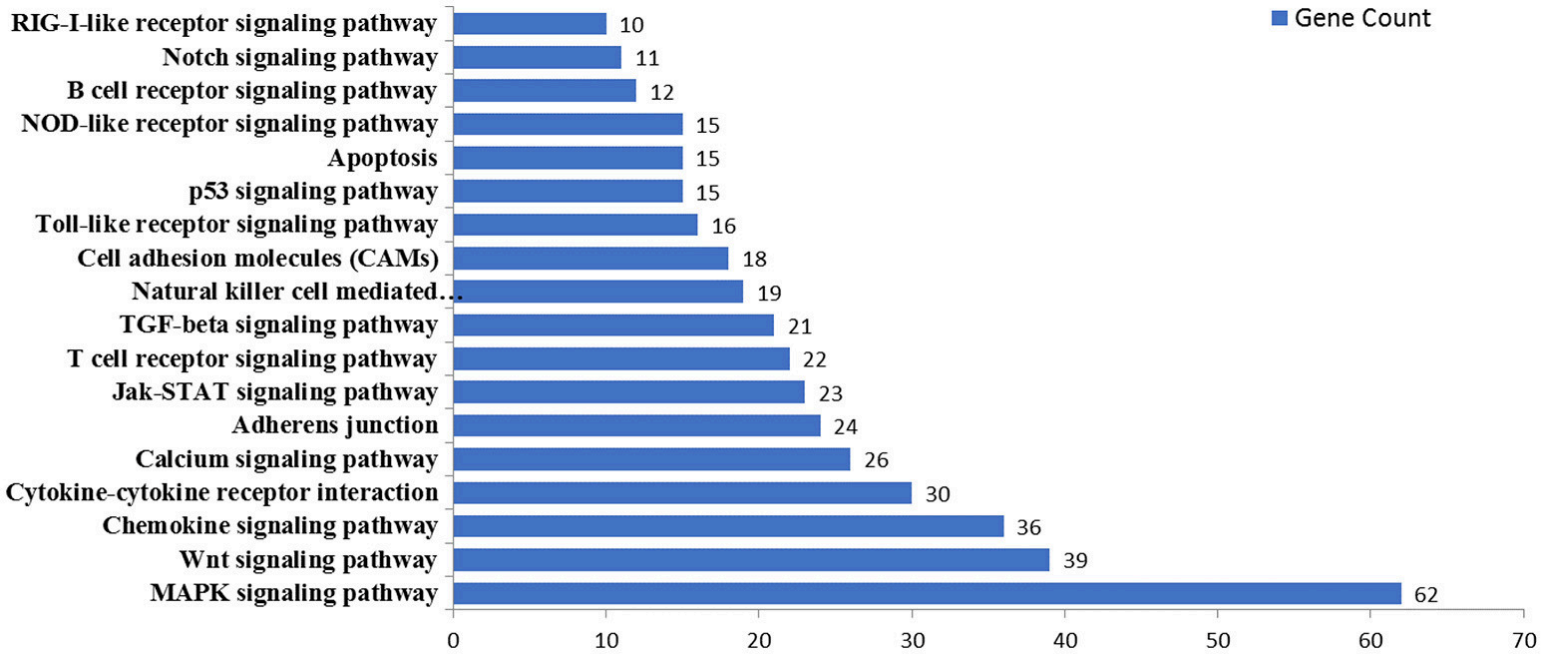
KEGG pathway analysis of predicted targets of differentially expressed miRNAs. The potential targets of four selected miRNAs were predicted by using TargetScan and MicroCosm. These predicted targets were further analyzed by using DAVID version 6.7. The number of predicted targets in 18 pathways associated with IAV replication were shown.

MicroRNAs function by binding to the 3′UTRs of their target genes, resulting in mRNA degradation or inhibiting mRNA translation (Bushati and Cohen, 2007). To identify target genes of these differentially expressed miRNAs during IAV infection, two computational algorithm programs, TargetScan and MicroCosm, were used to predict the target genes. The informatics and systems biology tool, DAVID database, was then used to determine the potential relationship of the target genes. After the cut-off criteria of FDR < 1 and *P* < 0.01, GO analysis indicated that 14 GO terms of molecular function were involved in IAV, such as transcription regulator activity and protein kinase activity (Table 1, Table S2). In addition, 18 pathways associated with IAV replication were chosen for further detailed analysis based on the DAVID database. We found at least 10 target genes were involved in any of these pathways (Figure 5, Table S2). For example, 10 correlated target genes were involved in the RIG-I signaling pathway, while 62 target genes were involved in the MAPK signaling pathway. Together, these results supported that the targets of IAV-regulated miRNAs were important for the regulation of cellular defenses during IAV infection.

**Table 1:** GO analysis of the predicted targets of differentially expressed miRNAs^a^.

**GO Id**	**Name**	**Count**	***P*-value**	**Fold enrichment**
GO:0030528	Transcription regulator activity	331	1.97E-12	1.414019
GO:0003700	Transcription factor activity	219	2.33E-09	1.450837
GO:0008134	Transcription factor binding	128	1.72E-08	1.611653
GO:0016563	Transcription activator activity	106	4.92E-08	1.669941
GO:0004672	Protein kinase activity	136	3.90E-06	1.44959
GO:0043565	Sequence-specific DNA binding	136	4.25E-06	1.447202
GO:0004674	Protein serine/threonine kinase activity	100	1.91E-05	1.50214
GO:0003677	DNA binding	427	2.89E-05	1.183218
GO:0000287	Magnesium ion binding	102	5.66E-05	1.457608
GO:0003702	RNA polymerase II transcription factor activity	62	6.60E-05	1.641273

a *The predicted targets were subjected to GO analysis by using DAVID version 6.7 and top 10 significantly GO terms of molecular function were shown in this table*.

### miR-21-3p directly targets the 3′UTR of HDAC8

**Figure 6:**
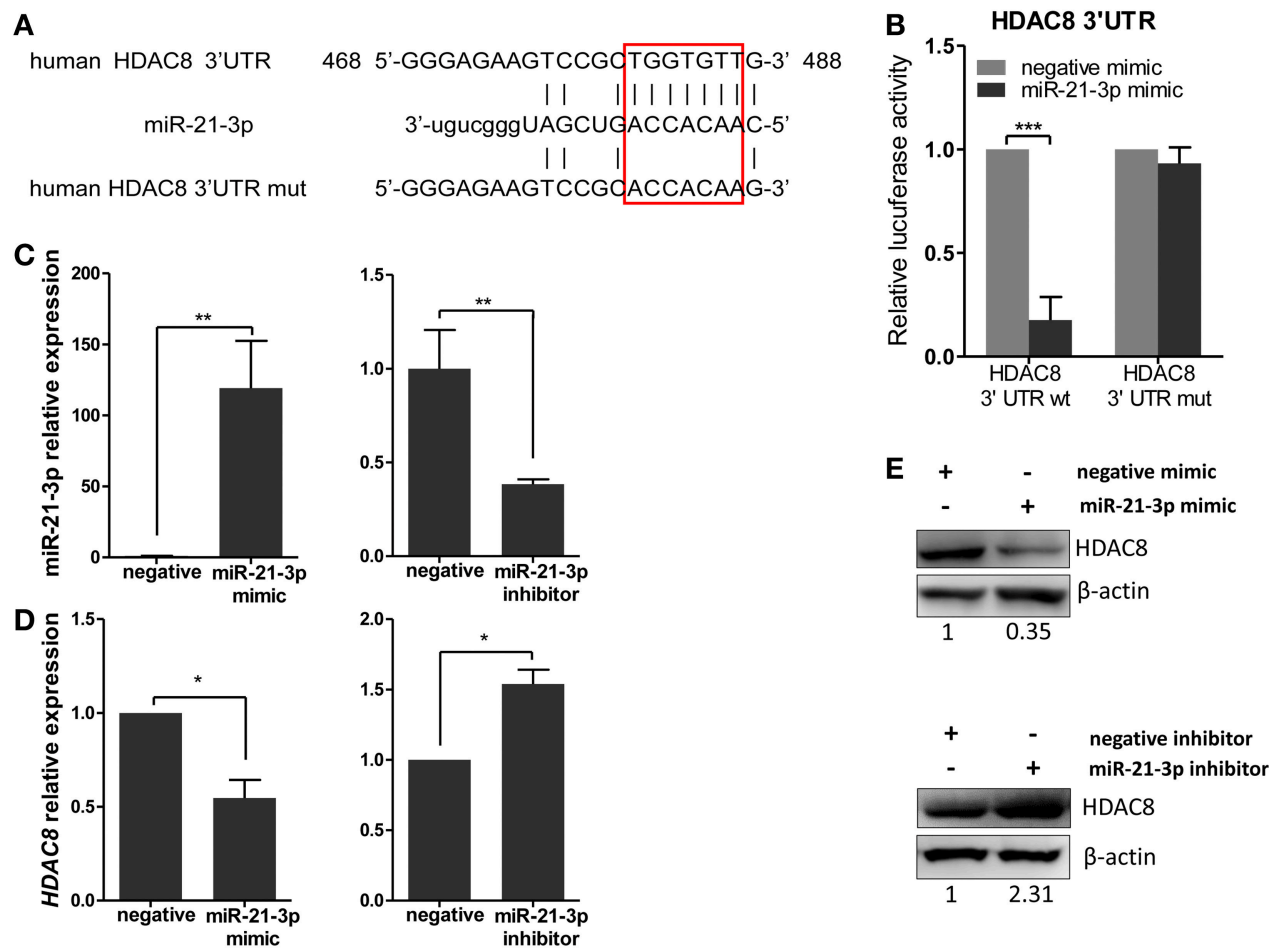
miR-21-3p directly targets the 3′UTR of HDAC8. **(A)** Schematic representation of predicted target sites of miR-21-3p in the 3′UTR of *HDAC8* and mutant *HDAC8* 3′UTR reporter constructs. **(B)** 293FT cells were co-transfected with pMIR-HDAC8-3′UTR luciferase reporter plasmid, pMIR-HDAC8-3′UTR mut luciferase reporter plasmid and pRL-TK plasmid, together with negative mimic or miR-21-3p mimic (50 nM). After 24 h, firefly luciferase activity was measured and normalized by Renilla luciferase activity. Data are the mean ± SD from four independent experiments. ^***^P < 0.001 vs. negative mimic by *t*-test. **(C)** A549 cells were transfected with miR-21-3p mimic or negative mimic (left), miR-21-3p inhibitor or negative inhibitor (right) at a final concentration of 50 and 100 nM, respectively. After 24 h, miR-21-3p expression was measured. Data are the mean ± SD of triplicate samples from a single experiment and are representative of two independent experiments. ^**^P < 0.01 vs. negative control by *t*-test. **(D)** A549 cells were transfected with mimics or inhibitors of miR-21-3p and their negative control. After 24 h, the *HDAC8* mRNA level was detected by real-time qPCR and normalized to expression of *GAPDH*. Data are the mean ± SD from three independent experiments. ^*^P < 0.05 vs. negative control by *t*-test. **(E)** A549 cells were transfected with mimics or inhibitors of miR-21-3p and their negative control. After 48 h, HDAC8 protein expression was analyzed by Western blot. β-actin was used as loading control. Data are representative of three independent experiments.

To further analyze these targets, HDAC8, a member of the HDAC family, relevant to IAV replication, was examined in greater detail. By using the bioinformatics tool, MicroCosm, we found the target site of miR-21-3p in the 3′UTR region of the human *HDAC8* gene (Figure 6A). To confirm the miRNA binding site and function in the *HDAC8* gene, the full-length 3′UTR of *HDAC8* was subcloned downstream of the luciferase reporter gene in the pMIR-Reporter vector. the mutant plasmid, pMIR-HDAC8-3′UTR mut, was also generated (Figure 6A). By co-transfection of the reporter plasmids and pRL-TK plasmid together with miRNA mimics in 293FT cells, the miR-21-3p mimic was shown to significantly reduce the luciferase activity of cells transfected with the wild-type pMIR-HDAC8-3′UTR plasmid. However, the luciferase activity was not reduced in cells transfected with the 3′UTR mutant plasmid (Figure 6B). Furthermore, transfection of the miR-21-3p mimic decreased HDAC8 expression in A549 cells at the levels of mRNA and protein, whereas the miR-21-3p inhibitor increased HDAC8 expression (Figures 6C–E). Together, these results suggested that miR-21-3p directly targeted HDAC8 through binding its 3′UTR and regulated the expression of endogenous HDAC8 via both mRNA degradation and translational inhibition.

### miR-21-3p promotes IAV replication through HDAC8

**Figure 7:**
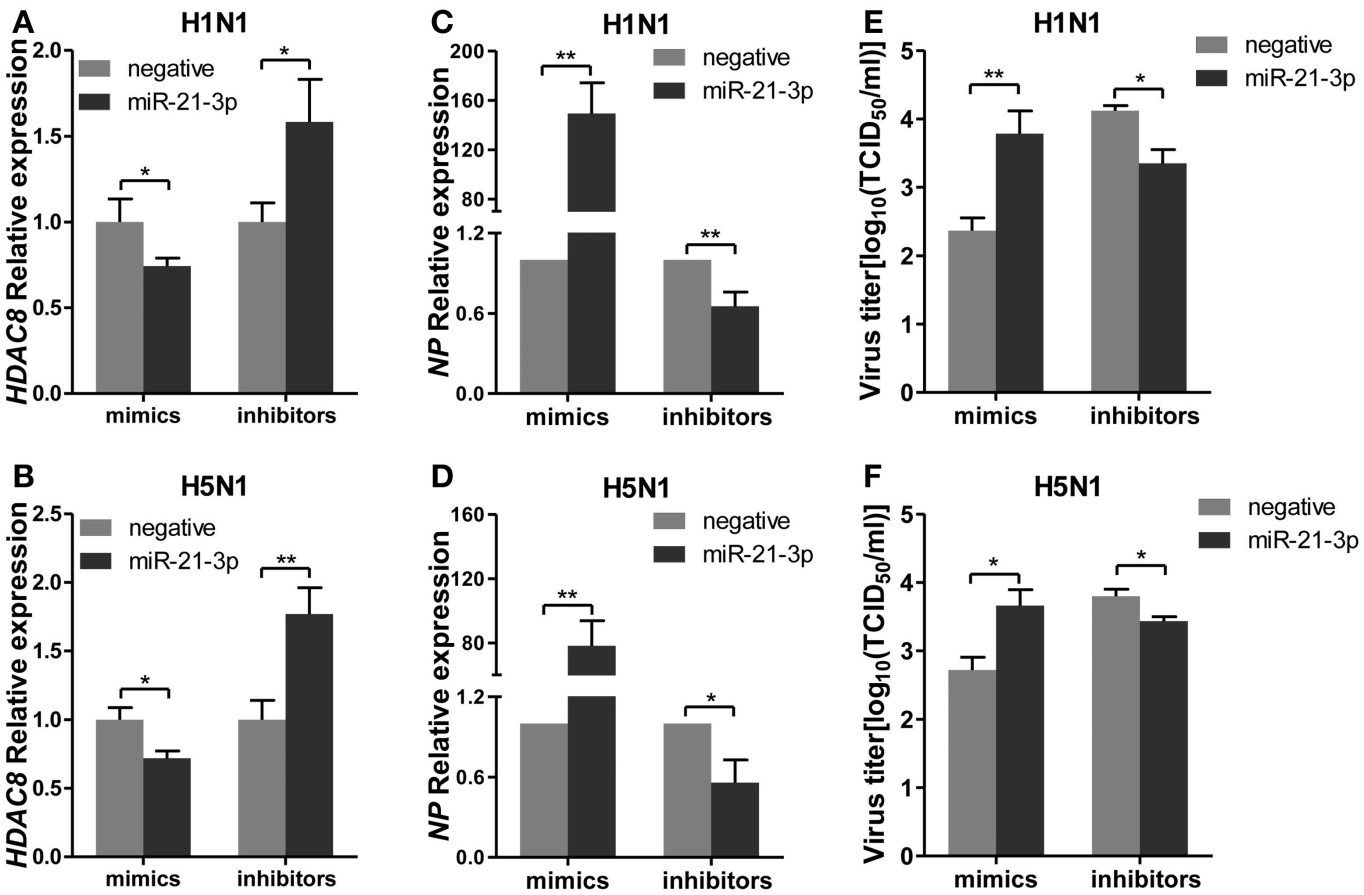
miR-21-3p promotes IAV replication. A549 cells plated in 24-well plates were transfected with mimics or inhibitors of miR-21-3p and their negative control (mimics: 50 nM; inhibitors: 100 nM). Twenty-four hours after transfection, cells were infected with H1N1 or H5N1 at a MOI of 0.1. **(A,B)** After 48 hpi, the relative *HDAC8* mRNA levels were analyzed by real-time qPCR and normalized to that of *GAPDH*. Data are the mean ± SD of triplicate samples from a single experiment and are representative of two independent experiments. ^*^P < 0.05 and ^**^P < 0.01 by *t*-test. **(C,D)** Viral *NP* vRNA was analyzed at 48 hpi by real-time qPCR. Data are the mean ± SD from four independent experiments. ^*^P < 0.05 and ^**^P < 0.01 vs. negative control by *t*-test. **(E,F)** Virus titers in supernatants were measured at 48 hpi by the TCID50 assay performed on MDCK cells. Viral titers are the mean ± SD from at least three independent experiments. ^*^P < 0.05 and ^**^P < 0.01 vs. negative control by *t*-test.

**Figure 8:**
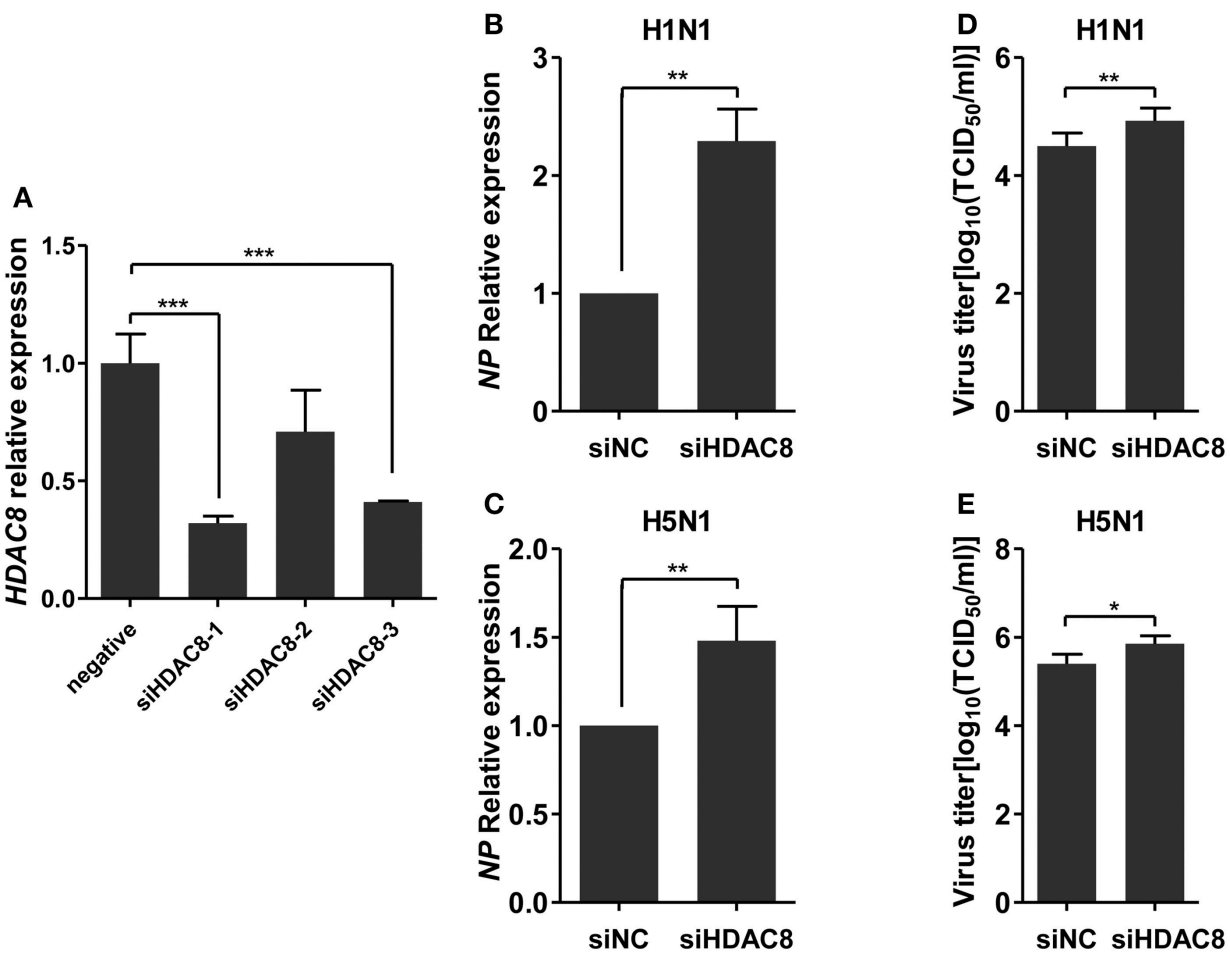
Knockdown of HDAC8 enhances IAV replication. **(A)** A549 cells were transfected with three different HDAC8 siRNAs (siHDAC8-1, siHDAC8-2, siHDAC8-3) or a negative control siRNA (siNC) at a final concentration of 25 nM. After 48 h, relative *HDAC8* mRNA levels were measured by real-time qPCR and normalized to that of *GAPDH*. Data are the mean ± SD of triplicate samples from a single experiment and are representative of two independent experiments. ^***^P < 0.001 by *t*-test. **(B–E)** A549 cells were transfected with siHDAC8-1 or siNC at a final concentration of 25 nM. Twenty-four hours after transfection, cells were infected with H1N1 or H5N1 at a MOI of 0.1. Viral *NP* vRNA was analyzed at 48 hpi by real-time qPCR **(B,C)**, and virus titers in supernatants were measured by the TCID50 assay performed on MDCK cells **(D,E)**. Data are the mean ± SD from four independent experiments. ^*^P < 0.05 and ^**^P < 0.01 vs. negative control by *t*-test.

To further explore the biological significance of the down-regulated miR-21-3p expression, we tested the effects of miR-21-3p on IAV replication. Subsequently, we infected A549 cells with IAV (H1N1 or H5N1) at a MOI of 0.1 in the presence of miR-21-3p mimic or inhibitor and then quantified the viral load. As shown in Figures 7A,B, the expression of *HDAC8* was decreased by the miR-21-3p mimic and up-regulated by the miR-21-3p inhibitor. Viral RNA quantified by measuring the *NP* gene expression was increased in infected cells in the presence of the miR-21-3p mimic, while the miR-21-3p inhibitor significantly decreased the amount of *NP* in infected cells (Figures 7C,D). Similar results were also found by measuring IAV TCID50 in the supernatants of infected cells (Figures 7E,F). Furthermore, the roles of HDAC8 in IAV replication were verified by an RNAi strategy. Three HDAC8-specific siRNAs were designed, and the siRNA of siHDAC8-1 was chosen for further study (Figure 8A). As shown in Figures 8B–E, the amount of *NP* and TCID50 increased in infected cells transfected with siHDAC8-1, which was consistent with the effect of the miR-21-3p mimic. However, we found that the overexpression of HDAC8 did not appear to significantly influence IAV replication at 48 hpi (Figure S4), indicating that the expression of exogenous HDAC8 is insufficient to promote IAV replication in A549 cells. Together, these results demonstrated that miR-21-3p could promote IAV replication, at least in part, via regulation of HDAC8.

## Discussion

MicroRNAs, as multifunctional regulators, have been widely used to study host-virus interactions (Skalsky and Cullen, 2010). To regulate virus replication, miRNAs may target host genes required for the virus life cycle or viral genes (Song et al., 2010; Ma et al., 2012; Nakamura et al., 2015; Zhou et al., 2015). A549 cells, a cell line of human lung epithelial carcinoma, are particularly sensitive to IAV infection and have been widely used as a good *in vitro* model to study IAV for nearly 20 years (Huang and Turchek, 2000). Here, we used miRNA profiling to investigate the differentially expressed miRNAs during H1N1 and H5N1 infection in A549 cells and found that a subset of four miRNAs, including miR-141, miR-200c, miR-21-3p, and miR-29b-1-5p, was dysregulated at both 8 hpi and 24 hpi. In recent years, several studies have also indicated miRNAs expression could be regulated during IAV infection (Li et al., 2010; Buggele et al., 2012; Loveday et al., 2012; Lam et al., 2013). However, there was little commonality or differences in direction of regulation among the affected miRNAs during IAV infection in these studies. Several potential reasons may account for the differences among these studies, such as the host genetic background, subtypes of IAV and heterogeneous endogenous tissues. Fortunately, in our study, two of the four miRNAs, miR-200c, and miR-21-3p, were consistent in their direction of regulation during IAV infection (Buggele et al., 2012; Lam et al., 2013). Interestingly, we found that avian IAV could more strongly regulate cellular miRNAs, compared to seasonal human IAV- (Lam et al., 2013), which may be attributed to differences in virulence of the two subtypes of IAV and should be confirmed in future studies.

Some miRNAs could be nonspecifically regulated by viral mimics (Buggele et al., 2012; Rosenberger et al., 2012). In this study, we indicated that UV-treated IAV and a synthetic mimetic of viral double-stranded RNA, poly(I:C) could not change the expression of the four miRNAs in A549 cells (Figures 3D, 4C). These findings showed that viral replication was essential for the regulation of the expression of those miRNAs. miRNAs regulate the expression of genes by inducing mRNA degradation or inhibiting mRNA translation through binding to the 3′UTRs of their target genes. Based on this characteristic, hundreds of potential targets were identified for each miRNA, which regulate cellular physiology through different mechanisms. Subsequently, GO and pathway analysis revealed that the targets of these miRNAs were involved in fundamental cellular pathways and pathways associated with IAV replication, such as the MAPK and Wnt signaling pathways. Some potential targets of these miRNAs identified in this study have been reported in responses to virus. For example, Ho et al. (2011) reported that an enterovirus could stimulate miR-141 expression and inhibit the host protein translation by targeting eIF4E to increase viral propagation. Another recent report showed that miR-141 and miR-200c potentially targeted Sdc2, which is involved in HSV-1 cellular attachment and entry (Majer et al., 2017).

Most studies have focused on the function of up-regulated miRNAs during IAV infection. However, we chose miR-21-3p, which was down-regulated in human lung epithelial carcinoma cells (Figure 2E) for further study. miR-21-5p (also known as miR-21) is one of the best studied miRNAs, and plays an important role in many cancers and viral infection when aberrantly expressed (Damania et al., 2014; Fu et al., 2015). miR-21-5p and the passenger strand (miR-21-3p) are derived from the same RNA precursor, pre-miR-21. Traditionally, the guide strand is involved in gene regulation, while the passenger strand is considered to be degraded (Guo and Lu, 2010). Recently, some reports demonstrated that many miRNA^*^ species are relatively rich and play important roles in various processes. Byrd et al. (2012) revealed that a passenger strand miRNA, miR-30c-2^*^, was induced by the unfolded protein response (UPR) and limited expression of proadaptive factor XBP1. Jin et al. (2011) reported that miR-149^*^ was directly induced by p53 and targeted GSK3α, which resulted in up-regulation of Mcl-1 and resistance to apoptosis in melanoma cells. In our study, we found that the expression of miR-21-3p in A549 cells was relatively enriched (Figure S2). However, the function of the passenger strand miRNA, miR-21-3p, in A549 cells during IAV infection is still unclear.

In the present study, we demonstrated that miR-21-3p promoted IAV replication. Recently, some target genes of miR-21-3p were identified to be involved in physiological and pathological processes. Báez-Vega et al. (2016) demonstrated that miR-21-3p inhibited proliferation and invasion of ovarian cancer cells by targeting RBPMS. Herein, we chosen ten potential targets of miR-21-3p for further analysis. Following luciferase assays, eight candidate genes were confirmed as targets of miR-21-3p, playing important roles in IAV infection. For example, FOXO3 was described as a transcription factor that negatively regulated the expression of proinflammatory cytokines and was modulated by H5N1 and H7N9 to induce cytokines production (Dejean et al., 2009; Ye et al., 2015). BIRC3/cIAP2 is a member of the family of cellular inhibitor of apoptosis proteins (cIAPs), which are involved in various cellular signaling pathways including cell death, cell cycle and immunity (Dubrez-Daloz et al., 2008). Rodrigue-Gervais et al. reported that cIAP2 promoted host survival during IAV infection by preventing pulmonary tissue necrosis (Rodrigue-Gervais et al., 2014).

Among these eight target genes, miR-21-3p showed the strongest inhibitory effect on the luciferase activity of the pMIR-report vector linked to HDAC8 3′UTR (Figure S3). In addition, using the miR-21-3p mimic and inhibitor, we found miR-21-3p could regulate HDAC8 expression at the both levels of mRNA and protein (Yan et al., 2015). HDAC8 is a member of the HDAC family of enzymes, which catalyze the deacetylation of acetylated proteins and regulate gene expression (Chakrabarti et al., 2015). The identification of more than 3,600 acetylation sites on 1,750 protein identified by proteomic analysis indicate that the regulatory scope of lysine acetylation is broad (Choudhary et al., 2009). Currently, at least 18 different HDACs have been found from mammalian cells and are grouped into four classes, including Class I HDACs (HDAC 1, 2, 3, and 8), Class IIa HDACs (HDAC 4, 5, 7, and 9), Class IIb HDACs (HDAC 6 and 10), Class III HDACs (SIRT1-7), and Class IV HDACs (HDAC11). HDACs play important roles in cancer, viral infection, neurological diseases and immune disorders (Falkenberg and Johnstone, 2014; Chakrabarti et al., 2015). In recent years, several studies have indicated that HDAC6 and HDAC1 could inhibit IAV progeny release and down-regulate IAV replication (Husain and Cheung, 2014; Nagesh and Husain, 2016). In this study, consistent with results using the miR-21-3p mimic, the replication of IAV was up-regulated in A549 cells by using siHDAC8 to knockdown HDAC8. However, we found that the effect of siHDAC8 on IAV replication was not greater than that of the miR-21-3p mimic, which implies that miR-21-3p acts as a multifunctional regulator in biological processes. A previous study reported that depletion of HDAC8 decreased the efficiency of IAV X31 entry using a microscopy-based assay (Yamauchi et al., 2011), which appeared to be inconsistent with our results. However, that study only monitored IAV entry in HDAC8-depleted cells using a microscopy-based assay and did not determine IAV titers by monitoring TCID50. In addition, two reports revealed that HDAC6, a member of the HDACs family, was essential for IAV entry, but it also inhibited IAV progeny release (Banerjee et al., 2014; Husain and Cheung, 2014). Thus, HDACs may have the opposite function in IAV infection and should be further investigated.

In summary, we performed miRNA profiling of A549 cells during IAV infection. Four miRNAs were identified to be responsive to IAV infection, especially H5N1, at both 8 and 24 hpi, and bioinformatic analysis revealed that the miRNAs were important regulators during IAV infection. However, more experiments need to be performed to confirm their biological functions. In this study, miR-21-3p was chosen for further analysis, and we found that it was down-regulated in A549 cells during IAV infection and could promote IAV replication at least partially through repressing HDAC8 expression. These results suggest that the host may resist IAV through down-regulation of miR-21-3p. Although data obtained from cultured cells cannot completely represent the consequences of infections *in vivo*, the findings here may still provide a perspective for the potential use of miRNAs in the treatment of viral infections.

## Author contributions

PH, XZ, and BX conceived and designed the experiments. BX, JL, XZ, and ZY performed the experiments. BX and XZ analyzed the data. RW and JL contributed reagents, materials, analysis tools. BX wrote the manuscript. All authors reviewed the manuscript.

### Conflict of interest statement

The authors declare that the research was conducted in the absence of any commercial or financial relationships that could be construed as a potential conflict of interest. The reviewer JY and handling Editor declared their shared affiliation.

## Supplementary material

Figure S1 Determination of CPE of A549 cells infected with IAV at different time points. A549 cells were infected with H1N1 and H5N1 at a MOI of 5 and CPE of infected cells was observed at different time points by using Nikon microscope and Canon Camera. Magnification, × 20.

Figure S2 miR-21-3p relative expression in A549 cells. Total RNA was purified from A549 cells and reverse transcribed using the First-Strand cDNA Synthesis Kit with a specific stem-loop primer. The expression of miR-21-3p and miR-200c were measured by qPCR and normalized to expression of *U6* snRNA. The expression of miR-200c was set to a value of 1 for normalization purposes. Data are the mean ± SD from four independent experiments. n.s., not significant, vs. miR-200c by *t*-test.

Figure S3 Analysis of miR-21-3p potential targets. **(A–H)** 293T cells were cotransfected with pRL-TK plasmid and luciferase reporter plasmids encoding the 3′UTRs of candidate genes, together with negative mimic or miR-21-3p mimic. After 24 h, firefly luciferase activity was measured and normalized by Renilla luciferase activity. Data are the mean ± SD from three independent experiments. ^*^*P* < 0.05, ^**^*P* < 0.01, and ^***^*P* < 0.001 vs. negative mimic by *t*-test.

Figure S4 HDAC8 overexpression does not affect IAV replication. **(A)** A549 cells were transfected with pCMV-tag2B-HDAC8 plasmid and negative control plasmid. After 24 h, HDAC8 protein expression was detected by flag antibody. Data are representative of two independent experiments. **(B,C)** A549 cells were transfected with pCMV-tag2B-HDAC8 plasmid and negative control plasmid. Twenty-four hours after transfection, cells were infected with H1N1 or H5N1 at a MOI of 0.1. Viral *NP* vRNA was analyzed at 48 hpi by real-time qPCR. Data are the mean ± SD from four independent experiments. n.s., not significant, vs. negative control by *t*-test.

Table S1 Primers used in this study.

Table S2 (Microsoft Excel format). GO and KEGG pathway analysis of targets of dysregulated miRNAs during IAV infection.
