# Supplementary figures and images for: miR-21-3p Regulates Influenza A Virus Replication by Targeting Histone Deacetylase-8
Source: Front Cell Infect Microbiol. 2018 May 25;8:175. doi: 10.3389/fcimb.2018.00175 (PMC5981164; doi:10.3389/fcimb.2018.00175)

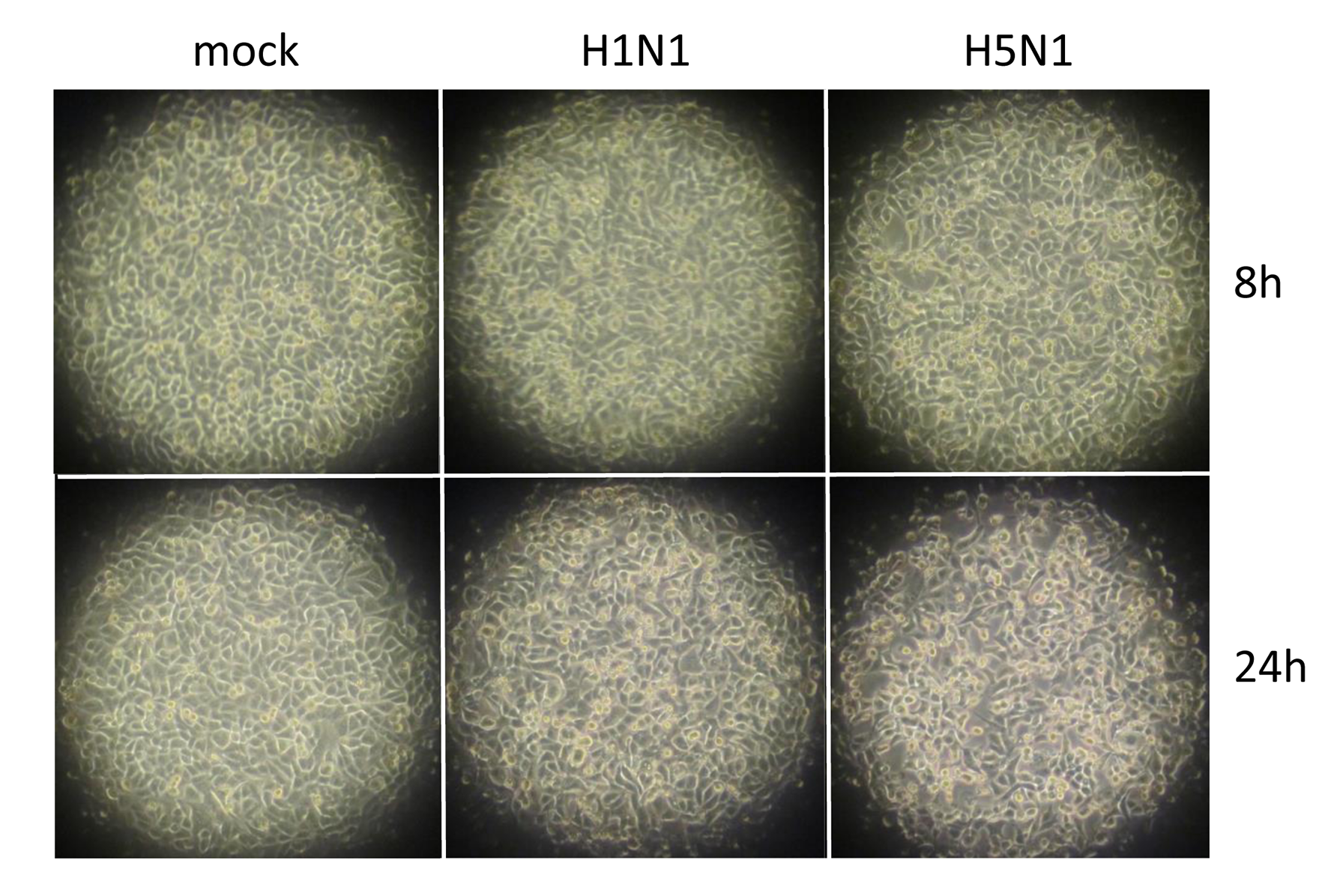

Supplement: Figure S1 — Determination of CPE of A549 cells infected with IAV at different time points. A549 cells were infected with H1N1 and H5N1 at a MOI of 5 and CPE of infected cells was observed at different time points by using Nikon microscope and Canon Camera. Magnification, × 20. [file Image_1.TIF]

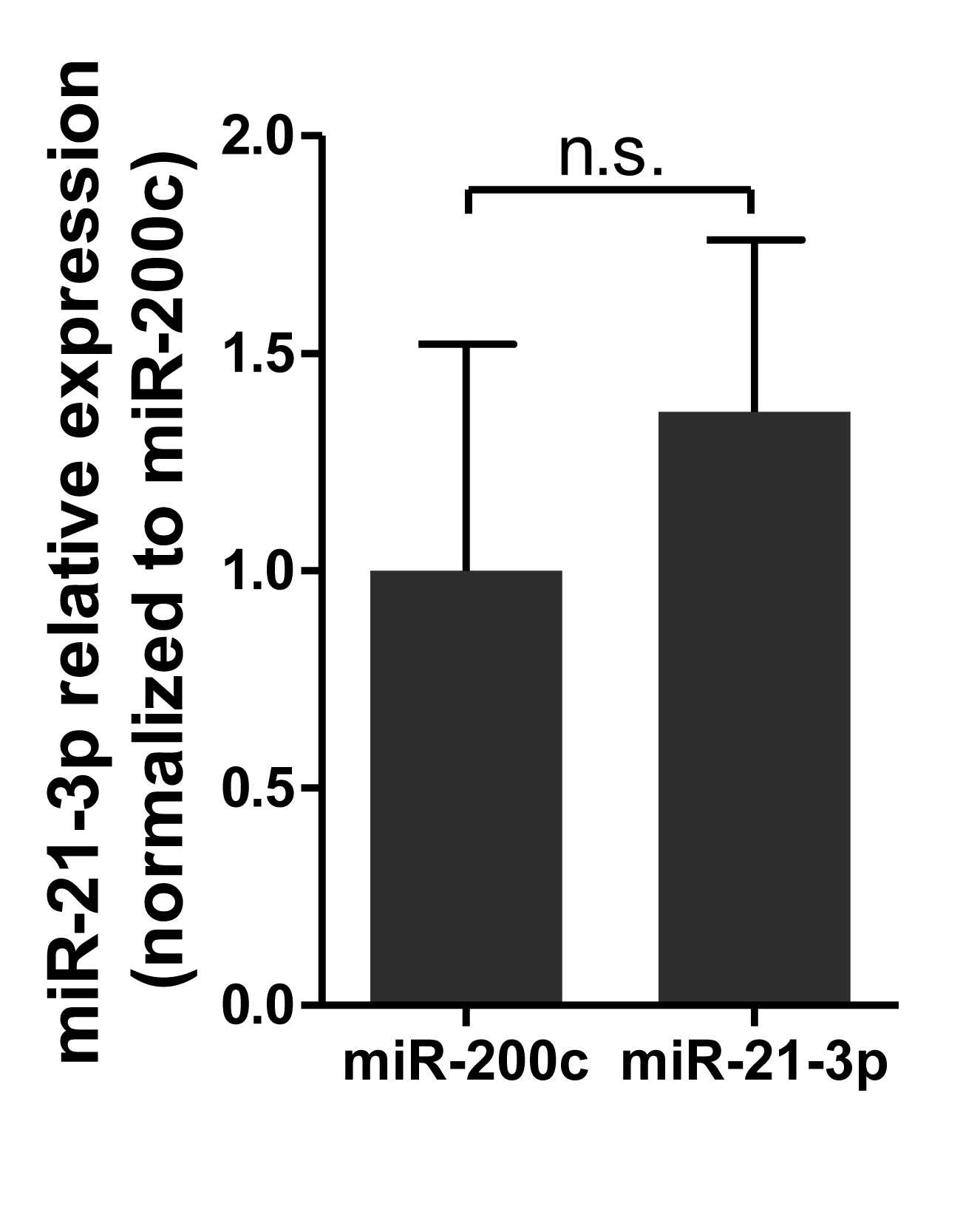

Supplement: Figure S2 — miR-21-3p relative expression in A549 cells. Total RNA was purified from A549 cells and reverse transcribed using the First-Strand cDNA Synthesis Kit with a specific stem-loop primer. The expression of miR-21-3p and miR-200c were measured by qPCR and normalized to expression of U6 snRNA. The expression of miR-200c was set to a value of 1 for normalization purposes. Data are the mean ± SD from four independent experiments. n.s., not significant, vs. miR-200c by t-test. [file Image_2.TIF]

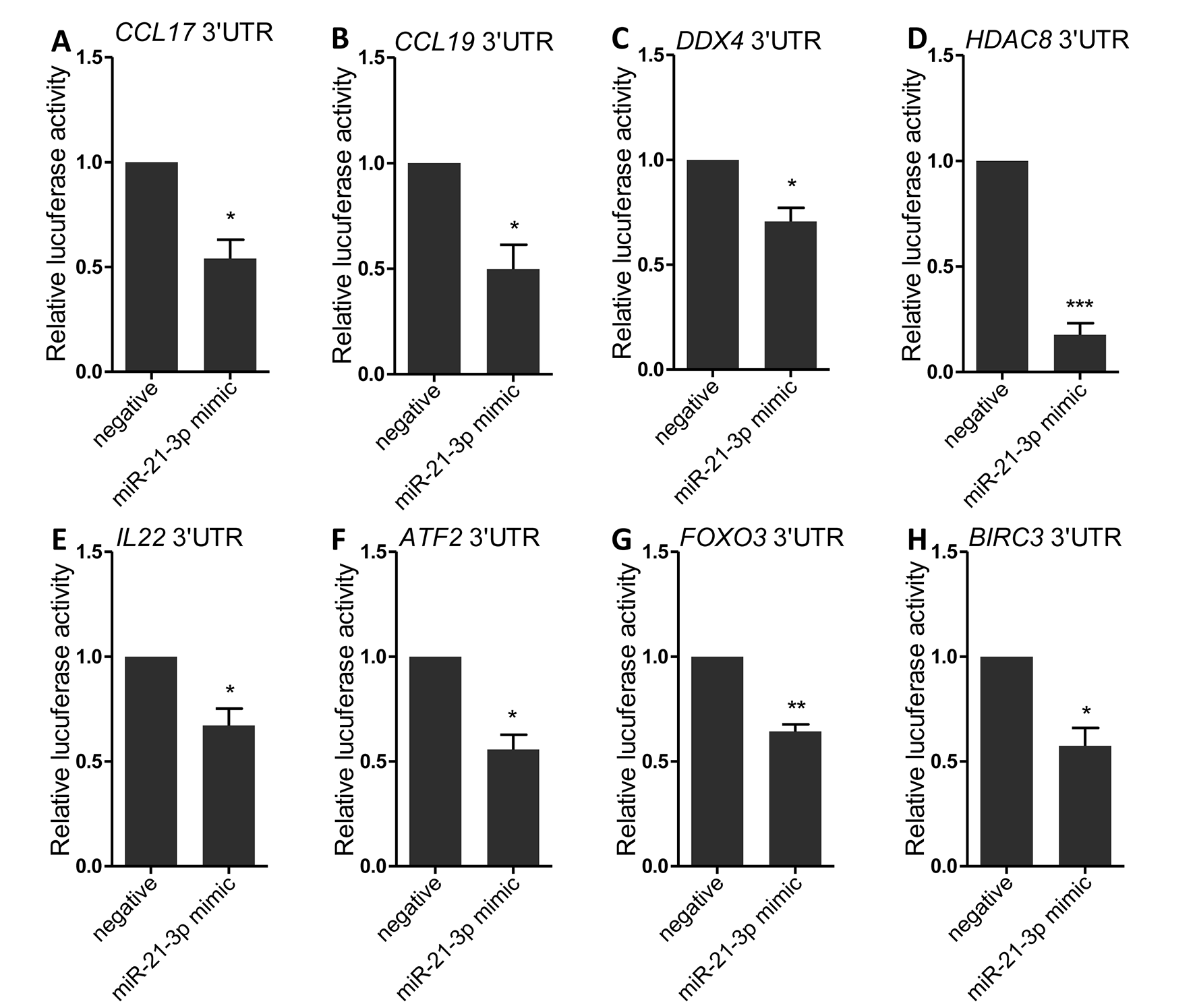

Supplement: Figure S3 — Analysis of miR-21-3p potential targets. (A–H) 293T cells were cotransfected with pRL-TK plasmid and luciferase reporter plasmids encoding the 3′UTRs of candidate genes, together with negative mimic or miR-21-3p mimic. After 24 h, firefly luciferase activity was measured and normalized by Renilla luciferase activity. Data are the mean ± SD from three independent experiments. *P < 0.05, **P < 0.01, and ***P < 0.001 vs. negative mimic by t-test. [file Image_3.TIF]

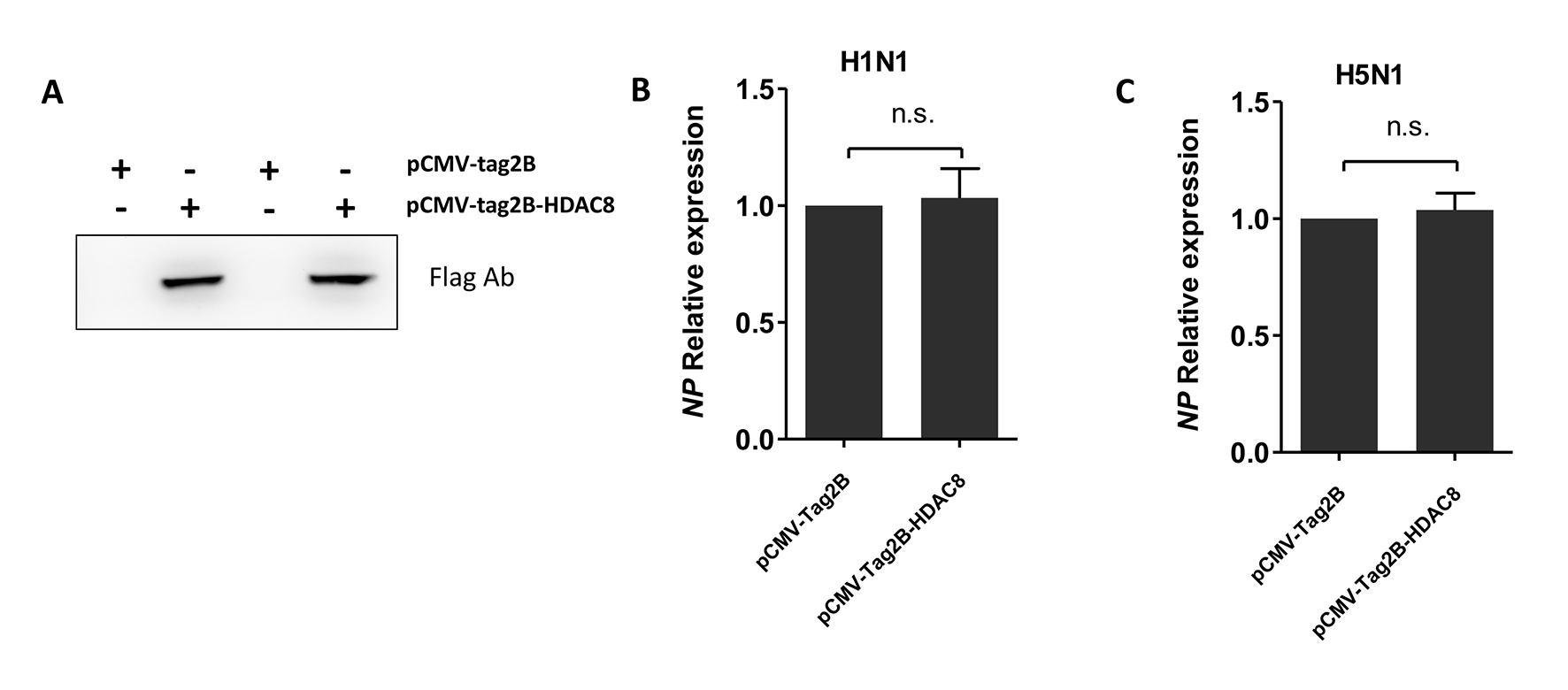

Supplement: Figure S4 — HDAC8 overexpression does not affect IAV replication. (A) A549 cells were transfected with pCMV-tag2B-HDAC8 plasmid and negative control plasmid. After 24 h, HDAC8 protein expression was detected by flag antibody. Data are representative of two independent experiments. (B,C) A549 cells were transfected with pCMV-tag2B-HDAC8 plasmid and negative control plasmid. Twenty-four hours after transfection, cells were infected with H1N1 or H5N1 at a MOI of 0.1. Viral NP vRNA was analyzed at 48 hpi by real-time qPCR. Data are the mean ± SD from four independent experiments. n.s., not significant, vs. negative control by t-test. [file Image_4.TIF]
