# Supplementary material for: miR-21-3p Regulates Influenza A Virus Replication by Targeting Histone Deacetylase-8
Source: Front Cell Infect Microbiol. 2018 May 25;8:175. doi: 10.3389/fcimb.2018.00175 (PMC5981164; doi:10.3389/fcimb.2018.00175)
Supplement: Table S1 — Primers used in this study. [file Table_1.DOCX]

**Table S1. Primers used in this study**

| name | Sequence (5'-3') |
| --- | --- |
| GAPDH-F | GCACCGTCAAGGCTGAGAAC |
| GAPDH-R | TGGTGAAGACGCCAGTGGA |
| HDAC8-F | GGTGACGTGTCTGATGTTGG |
| HDAC8-R | AGCTCCCAGCTGTAAGACCA |
| IFNbeta-F | GCTTGGATTCCTACAAAGAAGC |
| IFNbeta-R | ATAGATGGTCAATGCGGCGTC |
| CCL2-F | GATCTCAGTGCAGAGGCTCG |
| CCL2-R | TGCTTGTCCAGGTGGTCCAT |
| TNFalfa-F | GGCGTGGAGCTGAGAGATAAC |
| TNFalfa-R | GGTGTGGGTGAGGAGCACAT |
| H1N1-NP-F | GCCATAAGGACCAGGAGT |
| H1N1-NP-R | CGCTGAATGCTGCCATAA |
| H5N1-NP-F | CCAGAAGCGGAGGAAACA |
| H5N1-NP-R | GTCAAAGGAAGGCACGAT |
| HDAC8UTR-1F | CCGGAGCTCTTGACAGAAAGAGATCAGG |
| HDAC8UTR-1R | CCCAAGCTTTAGGTTTTCAAAGATTTTATTA |
| HDAC8UTR-1mF | GTGGGGAGAAGTCCGGACCACAACTTTTAGTGTTATATA |
| HDAC8UTR-1mR | TATATAACACTAAAAGTTGTGGTCCGGACTTCTCCCCAC |
